# Osteonecrosis of the Jaw in Post‐Bariatric Patients: An Underestimated Risk in Oral Surgery? Clinical Implications and Perspectives

**DOI:** 10.1111/scd.70108

**Published:** 2025-10-14

**Authors:** Wvelton Mendes Pereira, Ricardo Luiz Cavalcanti de Albuquerque‐Júnior, Pollianna Muniz Alves, John Lennon Silva Cunha

**Affiliations:** ^1^ Center of Biological and Health Sciences Federal University of Western Bahia (UFOB) Barreiras Brazil; ^2^ Department of Pathology Federal University of Santa Catarina (UFSC) Florianópolis Brazil; ^3^ Department of Dentistry State University of Paraíba (UEPB) Campina Grande Brazil

1

In recent years, the number of patients undergoing bariatric surgery has increased exponentially, driven by the growing prevalence of obesity [1, 2] and its comorbidities, such as hypertension, type 2 diabetes, and sleep apnea [3]. Roux‐en‐Y gastric bypass and sleeve gastrectomy are widely performed in young adults [3]. Consequently, oral health professionals, including dentists and dental specialists such as oral and maxillofacial surgeons, are increasingly likely to see patients undergoing these interventions. However, as this new patient profile becomes more common in the daily routine of dental clinics, a crucial question arises: could patients undergoing bariatric surgery have a higher risk of complications, such as osteonecrosis of the jaw (ONJ), after undergoing invasive oral surgical procedures? Although the delicate balance between nutrition, bone metabolism, and tissue healing may provide the underlying explanation based on pathophysiological reasoning, no specific clinical studies to date have systematically addressed this risk.

Bariatric surgeries, especially those with a malabsorptive component, such as Roux‐en‐Y gastric bypass, cause significant changes in the anatomy and physiology of the gastrointestinal tract, resulting in chronic compromise of nutrient absorption [4, 5]. Among the micronutrients most frequently affected are vitamin D, calcium, zinc, magnesium, iron, and B vitamins [4]. Vitamin D deficiency, for example, directly impacts calcium homeostasis, bone mineralization, and the regulation of the immune response [6, 7, 8]. Its deficiency, which often persists silently after surgery, can lead to hypocalcemia and the development of secondary hyperparathyroidism, favoring a state of chronic bone resorption [9]. Calcium, in turn, is essential for bone health and vital cellular functions such as muscle contraction, blood clotting, and intracellular signaling [10]. After bariatric surgery, calcium absorption is impaired due to a reduction in the absorptive surface area and a decrease in vitamin D activation within the body [11, 12]. In addition to affecting calcium homeostasis and bone mineralization, vitamin D deficiency also appears to impair implant osseointegration, as evidenced in both animal and human studies [13, 14, 15, 16, 17]. A recent systematic review has shown that vitamin D supplementation improves osseointegration in animals with systemic conditions, including vitamin D deficiency, osteoporosis, chronic kidney disease, and diabetes mellitus [17]. However, further investigations are needed to corroborate the hypothesis that humans derive similar benefits from vitamin D supplementation in terms of osseointegration.

In addition, other essential nutrients, such as zinc, magnesium, iron, and B vitamins, which play crucial roles in processes like angiogenesis, cell proliferation, inflammatory response, and tissue healing, are also compromised [13, 18, 19, 20, 21, 22, 23]. The deficiency of these elements significantly impairs tissue regeneration and the body's ability to face inflammatory and infectious challenges [13, 18, 19, 20, 21, 22, 23]. The major clinical concern is that these deficiencies often remain subclinical, with no evident manifestations for long periods [4]. Bariatric surgery has also been associated with a range of oral manifestations, including mucosal ulcers, dental erosion, caries, periodontal disease, xerostomia, and burning mouth syndrome [24, 25, 26].

Recently, Awang and colleagues reported the case of a 61‐year‐old Malaysian woman who developed denosumab‐induced hypocalcemia, with her history of bariatric surgery being cited as an aggravating factor [27]. Although antiresorptive drugs, such as denosumab and bisphosphonates, are recognized for their association with cases of ONJ [28], it is essential to note that post‐bariatric patients might be at an increased risk even in the absence of these medications. However, this remains a theoretical consideration, and further studies are warranted to determine whether the potential increased susceptibility has clinical relevance. Bariatric surgery itself, by causing chronic deficiencies of calcium, vitamin D, magnesium, and other micronutrients, compromises bone healing and could potentially predispose patients to tissue necrosis, including in procedures generally considered low risk, such as simple tooth extractions. Furthermore, in patients with post‐bariatric osteopenia or osteoporosis, a common condition in this group, the use of these drugs may further amplify the theoretical risk, especially when the nutritional and bone status is not previously investigated [27]. Therefore, even in the absence of exposure to medications known to be associated with ONJ, adopting more cautious clinical conduct in these individuals becomes essential. Although oral surgical procedures are generally considered low‐risk, the metabolic changes observed in post‐bariatric patients may increase their susceptibility to complications. These risks can arise both in the immediate postoperative period, due to acute deficiencies, and in the long term, since chronic nutritional imbalances can persist for years without adequate supplementation. However, despite these potential risks, there is currently no conclusive evidence directly linking bariatric surgery to ONJ. Until further studies confirm the true impact of bariatric surgery on post‐surgical bone health, the management of these patients should be cautious. Therefore, both a preventive dental evaluation before surgery and ongoing clinical care post‐operatively should be encouraged until further studies clarify the true impact of bariatric surgery on post‐surgical bone health.

Evidence from studies with animal models reinforces the pathophysiological plausibility of this association between bariatric surgery and changes in bone metabolism and immunity. In rats undergoing partial gastrectomy (vertical), a significant reduction in bone mineral density was observed, accompanied by structural changes in the trabecular architecture, indicative of disorganized bone remodeling [29]. In another experimental study with mice undergoing a gastric bypass model, there was a significant decrease in serum levels of vitamin D and calcium [30], accompanied by an increase in parathyroid hormone (PTH) and enhanced bone resorption, particularly in the cortical and trabecular portions of the bones. These findings suggest that secondary hyperparathyroidism resulting from chronic malabsorption may be a key factor in the loss of bone mass observed after bariatric surgery [31]. However, findings in animal models suggest that bone defects may occur with limited changes in serum PTH [32] or persist even after correcting circulating PTH to levels comparable to those of control rats [33, 34].

Additional studies in zinc‐deficient rats have demonstrated significant delays in wound healing, with impaired collagen synthesis, cell migration, and blood vessel formation [35, 36], which is particularly concerning in surgical settings. Similarly, magnesium deficiency has been linked to osteoblast dysfunction and increased osteoclastic activity, thereby exacerbating bone fragility [37]. Furthermore, in experimental models in obese or insulin‐resistant mice, wound healing has been frequently impaired, with a prolonged inflammatory response [38, 39]. Although specific studies on wound healing in bariatric mice are scarce, existing evidence suggests that compromised nutritional status and immunological alterations after surgery could hypothetically exacerbate this condition, potentially predisposing individuals to infection and impaired tissue repair [35, 36, 37, 38, 39]. However, this remains a theoretical consideration rather than established clinical evidence.

These experimental data suggest and support the hypothesis that nutritional deficiencies induced by bariatric surgery have profound systemic effects, compromising not only bone integrity but also the body's capacity for inflammatory and healing responses. Thus, even in the absence of factors traditionally recognized for ONJ, such as the use of antiresorptive drugs [40], a history of bariatric surgery should be considered a potential risk marker and justifies the adoption of more cautious clinical approaches, including preoperative laboratory evaluation, correction of possible nutritional deficiencies, and interdisciplinary monitoring of these patients. In this scenario, it is becoming increasingly evident that dentists and oral and maxillofacial surgeons must consider the history of bariatric surgery as a possible clinical risk factor during the anamnesis and planning of any invasive procedure. A recent study has shown that the frequency of dental interventions tends to increase after bariatric surgery, likely reflecting changes in oral health status [41]. This recommendation is supported by a growing body of evidence suggesting that bariatric procedures increase skeletal fragility related to excessive bone loss [42, 43], contribute to immune alterations [44, 45], and impair the wound healing process due to nutritional deficiencies [46]. Therefore, the presence of a history of bariatric surgery, especially in cases involving procedures with a malabsorptive component, should trigger a warning sign regarding the possibility of underlying nutritional deficiencies that, although silent, can profoundly compromise tissue repair, bone remodeling, and inflammatory response mechanisms [4, 42, 43]. This risk assessment must be complemented by objective measures that allow a preventive and personalized approach.

Among the most relevant clinical measures is the request for laboratory tests before the procedure, including serum levels of vitamin D, calcium, alkaline phosphatase, and PTH, which can provide essential clues about the patient's bone metabolism status [28]. Early identification of potential abnormalities enables targeted therapeutic interventions, such as vitamin supplementation, correction of electrolyte imbalances, or closer monitoring by a multidisciplinary team [47]. In this context, preoperative referral for evaluation by a nutritionist or endocrinologist can be a decisive differentiator in minimizing post‐surgical complications. From a technical point of view, it is recommended that dentists and oral and maxillofacial surgeons adapt their surgical procedures for these patients, prioritizing, whenever possible, less invasive techniques with less bone and tissue trauma. Also, postoperative follow‐up should be conducted with greater vigilance, including more frequent clinical follow‐ups and rigorous guidance on early signs of infection or necrosis, to enable early intervention in the event of any deviation from the expected healing process.

In addition to the immediate practical implications, this discussion also reveals an essential gap in the current scientific literature. Despite the increasing number of patients undergoing bariatric surgeries [1, 2] and the recognized prevalence of nutritional disorders in this population [4, 5], a notable lack of data remains on the outcomes of invasive dental procedures in post‐bariatric patients. The absence of specific guidelines and evidence‐based protocols hinders clinical decision‐making and limits the implementation of standardized procedures.

In this sense, there is a clear opportunity for the development of studies that expand knowledge about this interface between bariatric surgery and oral and maxillofacial surgery. Retrospective cohort studies, for example, can contribute to identifying the prevalence of oral surgical complications in bariatric patients and allow comparisons with control populations. Well‐documented case reports, especially of ONJ in individuals without exposure to antiresorptive drugs but with a history of bariatric surgery, can raise valuable hypotheses and foster relevant clinical discussions. In addition, prospective studies, especially multicenter studies, that integrate laboratory data, detailed nutritional assessments, and imaging exams, can provide a broader and more accurate view of the predictive factors for complications in this population group.

In summary, due to the growing number of post‐bariatric patients, clinicians should consider a history of bariatric surgery as a potential risk factor for invasive procedures, even in the absence of antiresorptive medication use. Therefore, prior laboratory evaluation, nutritional assessment, the use of minimally invasive surgical techniques, and careful postoperative monitoring are necessary. Despite the pathophysiological plausibility, evidence on oral complications, especially ONJ, in this population remains scarce. Until robust data are available, treatment should focus on prevention and be personalized and cautious, prioritizing patient safety.

## Author Contributions

**Wvelton Mendes Pereira**: writing – original draft. **Ricardo Luiz Cavalcanti de Albuquerque Júnior**: review and editing of the original draft. **Pollianna Muniz Alves**: review and editing of the original draft. **John Lennon Silva Cunha**: conceptualization, supervision, and review of the original draft.

## Ethics Statement

The authors have nothing to report.

## Conflicts of Interest

The authors declare no conflicts of interest.

## Data Availability

No new data were created or analyzed in this study. This work is based entirely on previously published literature and publicly available sources, and therefore, no additional datasets are associated with this article.
